# Long-Term Effects of Prenatal Exposure to Undernutrition on Cannabinoid Receptor-Related Behaviors: Sex and Tissue-Specific Alterations in the mRNA Expression of Cannabinoid Receptors and Lipid Metabolic Regulators

**DOI:** 10.3389/fnbeh.2016.00241

**Published:** 2016-12-27

**Authors:** María T. Ramírez-López, Rocío Arco, Juan Decara, Mariam Vázquez, Patricia Rivera, Rosario Noemi Blanco, Francisco Alén, Raquel Gómez de Heras, Juan Suárez, Fernando Rodríguez de Fonseca

**Affiliations:** ^1^Departamento de Psicobiología, Facultad de Psicología, Universidad Complutense de MadridMadrid, Spain; ^2^Hospital Universitario de GetafeMadrid, Spain; ^3^Instituto de Investigación Biomédica de Málaga (IBIMA), Unidad de Gestión Clínica de Salud Mental, Hospital Regional Universitario de Málaga, Universidad de MálagaMálaga, Spain; ^4^Departamento de Biología Celular, Genética y Fisiología, Instituto de Investigación Biomédica de Málaga (IBIMA), Facultad de Ciencias, Universidad de MálagaMálaga, Spain

**Keywords:** maternal diet, CB1 receptor, β-oxidation, lipoproteins, lipogenesis, hypothalamus, liver, adipose tissue

## Abstract

Maternal malnutrition causes long-lasting alterations in feeding behavior and energy homeostasis in offspring. It is still unknown whether both, the endocannabinoid (eCB) machinery and the lipid metabolism are implicated in long-term adaptive responses to fetal reprogramming caused by maternal undernutrition. We investigated the long-term effects of maternal exposure to a 20% standard diet restriction during preconceptional and gestational periods on the metabolically-relevant tissues hypothalamus, liver, and perirenal fat (PAT) of male and female offspring at adulthood. The adult male offspring from calorie-restricted dams (RC males) exhibited a differential response to the CB1 antagonist AM251 in a chocolate preference test as well as increased body weight, perirenal adiposity, and plasma levels of triglycerides, LDL, VLDL, bilirubin, and leptin. The gene expression of the cannabinoid receptors *Cnr1* and *Cnr2* was increased in RC male hypothalamus, but a down-expression of most eCBs-metabolizing enzymes (*Faah, Dagl*α*, Dagl*β*, Mgll*) and several key regulators of fatty-acid β-oxidation (*Cpt1b, Acox1*), mitochondrial respiration (*Cox4i1*), and lipid flux (*Ppar*γ) was found in their PAT. The female offspring from calorie-restricted dams exhibited higher plasma levels of LDL and glucose as well as a reduction in chocolate and caloric intake at post-weaning periods in the feeding tests. Their liver showed a decreased gene expression of *Cnr1, Ppar*α, *Ppar*γ, the eCBs-degrading enzymes *Faah* and *Mgll*, the *de novo* lipogenic enzymes *Acaca* and *Fasn*, and the liver-specific cholesterol biosynthesis regulators *Insig1* and *Hmgcr*. Our results suggest that the long-lasting adaptive responses to maternal caloric restriction affected cannabinoid-regulated mechanisms involved in feeding behavior, adipose β-oxidation, and hepatic lipid and cholesterol biosynthesis in a sex-dependent manner.

## Introduction

Although, overnutrition is an important life style factor for the development of metabolic syndrome, obesity, cardiovascular disease and diabetes mellitus (Alberti et al., 2009), maternal undernutrition resulting in early life nutritional unbalance can be also related to the onset of long-term metabolic alterations observed later in life. This process has been called nutritional programming (Lucas, 1991; Hales and Barker, 2001; Gluckman and Hanson, 2004; Barker, 2007; Vaag et al., 2012). Importantly, this hypothesis supports that fetal undernutrition can represent the origin of cardiovascular disease, non-insulin-dependent diabetes mellitus, hypertension and hyperlipidaemia at adulthood (Barker and Osmond, 1988; Barker et al., 1989, 1993).

The epidemiological data from the Dutch Famine have showed that people whose mothers were exposed to undernutrition in early gestation developed more metabolic abnormalities than people exposed in other pregnancy stages (Roseboom et al., 2006). Currently, maternal undernutrition is a problem not exclusive of developing countries. The pressure to thinness in western societies associated to a higher prevalence of women with a past of eating disorders or a worry in excess about body changing during pregnancy and postpartum have raised the risk for giving birth underweight babies (Easter et al., 2013; Linna et al., 2014). However, we should not discard a predictive marker of impaired fetal nutrition in those cases with absence of low birth weight.

Experiments with animals models have succeeded in simulate metabolic alterations of undernutrition during gestational periods in humans, particularly by the implementation of a moderate or severe calorie restricted diets (Desai et al., 2005; García et al., 2010; Palou et al., 2010; Suzuki et al., 2010; Lukaszewski et al., 2011). Consequently, the exposure to a moderate or severe restricted diet during pregnancy has been associated to features of metabolic syndrome in adult offspring such as higher adiposity (Yura et al., 2005; Suzuki et al., 2010; Lukaszewski et al., 2011), alterations in glucose metabolism (Jimenez-Chillaron et al., 2005; Breton et al., 2009; Theys et al., 2011), or alterations in the lipid plasmatic profile (Desai et al., 2005, 2007a; Palou et al., 2010, 2012). Moreover, these metabolic alterations after a maternal calorie restricted diet have been found associated to abnormalities in feeding behavior such as hyperphagia (Vickers et al., 2000; Breton et al., 2009; Manuel-Apolinar et al., 2014) or high preference for high-fat diet (Palou et al., 2010; Lukaszewski et al., 2011). Some studies have also noticed that the metabolic profile exhibited by offspring depends on the sex (Desai et al., 2007a; Palou et al., 2010; Suzuki et al., 2010).

Several biologic mechanisms underlying the nutritional programming, which become altered after the exposure to a maternal restricted diet during pregnancy, have been identified. They include epigenetic regulation (Nijland et al., 2010; Martinez et al., 2014), leptin signaling (Yura et al., 2005; Palou et al., 2010), hypothalamic development (Sebert et al., 2009; García et al., 2010), and dopaminergic and serotonergic signaling systems (Manuel-Apolinar et al., 2014). Interestingly, recent studies have documented epigenetic modifications or affectation of adrenocortical growth in offspring after maternal dietary restriction around the time of conception (Nicholas et al., 2013; Zhang et al., 2013), pointing out the importance of the periconceptional window in the metabolic programming.

The endocannabinoid system (ECS) is also implicated in metabolic and behavioral mechanisms involved in fetal programming such as leptin signaling (Di Marzo et al., 2001) or dopaminergic system (Melis et al., 2004). The ECS keeps a homeostatic role in regulating energy balance and food intake (Cristino et al., 2014), and its overactivation, mainly via CB1 receptors, favors the energy accumulation, increases the appetite for highly palatable foods, decreases the satiety and reduces the energy expenditure. Long-term effects of ECS activation could finally increase the risk from suffering metabolic diseases that lead to obesity and metabolic syndrome (Tibirica, 2010; Cristino et al., 2014). Additionally, it has been revealed that some ECS components are sensible to dietary conditions. As the main endogenous agonists (anandamide and 2-arachidonoyl glycerol) of the cannabinoid receptors CB1 and CB2 are derivatives of fatty acids, it has been demonstrated that the lipid profile of the diet could modify the endocannabinoid levels (Artmann et al., 2008) in some tissues, including the early developing brain (Berger et al., 2001). Consequently, the cannabinoid receptor activity may be modified. Furthermore, the gene expression of the ECS components could be modified after the exposure to different dietary conditions (Bello et al., 2012). Taking together, these data suggest that the ECS could play a putative role in nutritional programming and hence in the early origin of metabolic diseases.

Regarding the potential relation between ECS and fetal undernutrition, a few studies have demonstrated that the maternal exposure to a calorie-restricted diet during pregnancy and/or lactation could decrease the hypothalamic endocannabinoids and/or acylethanolamines in offspring (Matias et al., 2003; Ramírez-López et al., 2016). However, it is unknown whether changes in nutritional programming by maternal diet restriction during fetal development could modify the expression of ECS components later in life and lead to long-lasting impact on energy metabolism and feeding behavior.

Based on these considerations, we aim to focus on the long-term effects on cannabinoid-related behaviors (i.e., cannabinoid receptor antagonist-induced suppression of feeding), and effects on lipid-related metabolic pathways of a maternal exposure to a moderate caloric restriction during the preconceptional and gestational period. Particularly, we evaluated the gene expression of relevant components of the endocannabinoid system, and key enzymes and regulators of the lipid and cholesterol metabolism in metabolically relevant tissues, such as the hypothalamus, the liver, and the perirenal adipose tissue (PAT), of male and female adult offspring. Additionally, it was assessed the spontaneous and compulsive feeding behavior, growing parameters, leptin, plasma metabolites, and adiposity after weaning (adolescence and adulthood). We hypothesize that the maternal exposure to a moderate calorie-restricted diet could alter the energy homeostasis regulated by the ECS and could re-program fatty-acid metabolism later in life in a sex-specific manner. We also proposed that these putative alterations could increase the vulnerability to develop metabolic diseases in adulthood.

## Materials and methods

### Ethics statement

Experimental procedures with animals were carried out following with the recommendations of the European Communities directive 2010/63/EU and Spanish legislation (Real Decreto 53/2013, BOE 34/11370-11421, 2013) regulating the care and use of laboratory animals. The protocol was approved by the Animal Ethics Committee of the Complutense University of Madrid.

### Animals, housing, mating, and feeding

**Figure 1:**
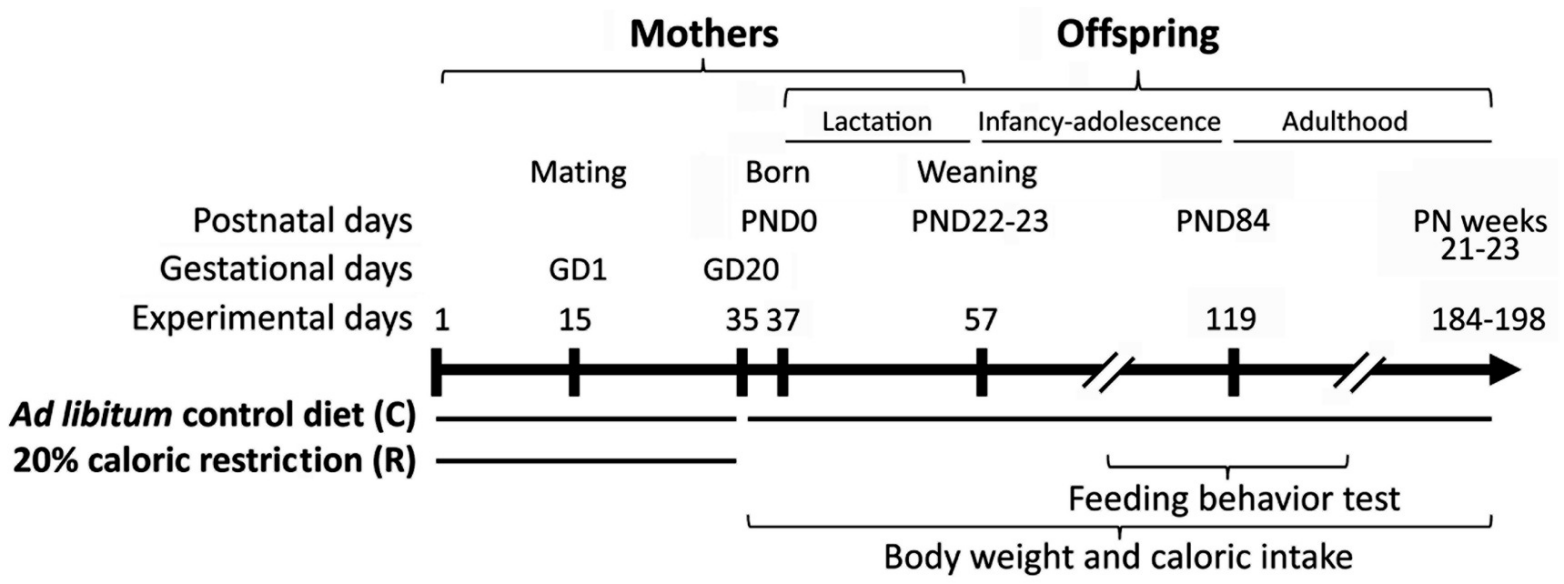
**Experimental design**. Two weeks before mating female rats were exposed to 20% caloric restriction up to the gestational day 20 (GD20). Body weight and caloric intake of offspring were monitored from birth (PND0) to adulthood (postnatal weeks 21–23). Feeding behavior (compulsive feeding and feeding response to AM251) was evaluated at adolescence (postnatal weeks 8–9) and adulthood (postnatal weeks 12–13).

Adolescent female Wistar rats (Harlan, Barcelona, Spain) weighing 191.7 ± 2.6 g were individually housed in standard cages and maintained in controlled room conditions at 21 ± 1°C room temperature, 40 ± 5% relative humidity and a 12-h light-dark cycle (lights off 8:00 p. m.). Animals were handled and allowed to acclimate for at least 4 weeks before the diet assignation. Then, 2 weeks before mating (Figure 1), animals were weighed (average weight 240.7 ± 3.4 g) and randomly exposed to an *ad libitum* access of a standard chow (control group; *n* = 9) or a restricted access of the same standard chow (caloric restriction group; *n* = 15). The females with caloric restriction were fed with a daily amount of food corresponding to the 80% of the calories that the control rats were fed in the previous day of the experiment, according to the body weight (20% of caloric restriction). The standard chow (Standard Chow SAFE A04, Panlab, Barcelona, Spain) contained 16.1% of protein, 60% carbohydrate, 3.1% fat, 4% fiber, and 0.0025% sodium, resulting in a 2.9 kcal/g as energy.

After 2 weeks of feeding (pregestational period), adult females (4 month old) were allowed to mate with male rats of the same strain weighting 484.5 ± 7.96 (Figure 1). The presence of plug or spermatozoa in the vaginal smear confirmed the successful mating, and this was designated as gestational day 0 (GD 0). Female rats were maintained on the same diet paradigm as in the pregestational period up to the gestational day 20 (GD 20). Thus, the caloric restriction finished at GD 20 (2 days before birth), and all dams and their offspring were fed *ad libitum* with the standard chow along the remaining experimental periods (lactation and postweaning periods). The day of birth was defined as postnatal day 0 (PND 0). Within the 14 h after birth, pups were weighed, sexed, and culled to comprise up to 8 pups per mother (4 males and 4 females where possible).

Food intake and body weight of the dams and pups were 3–4 days per week and/or weekly measured along lactation and post-weaning periods (infancy, adolescence, and adulthood). At PND 22–23, offspring were weaned, housed in groups (2–3 rats/cage) according to the litter and fed *ad libitum* with the same standard chow. Dams were sacrificed. Feeding behavior tests in the offspring were carried out at the adolescence (postnatal weeks 8–9) and the adulthood (postnatal weeks 12–13). At the postnatal weeks 21–23, adult offspring were finally weighted and sacrificed (Figure 1). In order to minimize the estrous cycle-related variability, the female rats were closely housed in adjacent cages and randomly distributed among the different experimental groups (McClintock, 1978, 1984). Moreover, males were housed in a separate room. We generated the following four experimental groups: Male offspring from control diet-fed dams (CC male; *n* = 15); Male offspring from calorie-restricted dams (RC male; *n* = 23); Female offspring from control diet-fed dams (CC female; *n* = 16); and Female offspring from calorie-restricted dams (RC female; *n* = 23). To avoid litter effects, samples from 6 to 15 liters per perinatal group were used in all determinations. The criteria described by Vickers were adopted to refer to different stages of development (Vickers et al., 2000).

### Measurement of caloric intake

Food intake was determined by subtracting the amount of food left in the cage from the total amount of food provided. To calculate individual food intake when animals were housed in groups, total food intake from each cage was measured and equally divided according to the number of pups per cage. Comparisons among groups were performed by calculating cumulative caloric intake relative to body weight (kcal/kg) at each time point.

### Compulsive feeding test

This test was an adaptation of the Compulsive Feeding Test described by Heyne et al. (2009). To assess the feeding behavior, rats were exposed to a free choice between a mixture of chocolates and a standard chow for limited and unlimited time. This test allows us to measure food intake, chocolate preference as well as to detect the development of inflexible feeding behavior. At the beginning of the test, the rats were individually housed and exposed to the two types of food (chocolate and standard chow) for 4 days. Rats had *ad libitum* access to the standard chow and water, and had limited access to the chocolate for 1 h during the light phase. The position of chocolate mixture was switched every day. At the end of the test, animals were returned to their original cages and standard diet. Total caloric intake relative to body weight and chocolate preference (calculated as the percentage of chocolate eaten on overall intake) were evaluated at adolescence (postnatal weeks 8–9) and reevaluated at adulthood (postnatal weeks 12–13) in all experimental groups.

### Test of feeding response to AM251

To evaluate the implication of the cannabinoid CB1 receptors in the differential response on caloric intake and chocolate preference, adult rats (postnatal weeks 13–14) were firstly treated with a dose of the CB1 receptor inverse agonist AM251 (3 mg/kg) and then exposed to a free choice between a mixture of chocolates and a standard chow. AM251 (Tocris, Bioscence, Bristol, UK) was dissolved in a vehicle containing 5% Tween 80 and 95% saline. The day before the test, all animals were food-deprived for 20 h. Then AM251 or vehicle was intraperitoneally administered (3 mg/mL). After 30 min, animals were placed in an individual cage without bedding material. Then, they were provided with two small cans containing both types of food that were previously weighed. Chocolate and total caloric intakes relative to body weight (kcal/kg) were calculated for 4 h. We generated the following eight experimental offspring groups: Vehicle in CC male (*n* = 7); AM251 in CC male (*n* = 8); Vehicle in RC male (*n* = 9); AM251 in RC male (*n* = 10); Vehicle in CC female (*n* = 6); AM251 in CC female (*n* = 7); Vehicle in RC female (*n* = 11); and AM251 in RC female (*n* = 11).

### Sample collection

At 21–23 postnatal weeks, adult offspring were sacrificed by decapitation after the administration of Equitesin® (3 mg/kg). This process was carried out in the 2 following hours after the beginning of the dark phase in a separate room from the other experimental animals. Blood samples were briefly collected into tubes containing EDTA (6%) and centrifuged (1500 *g* for 10 min at 4°C). The plasma was removed, frozen and stored at −80°C for biochemical and hormonal analysis. White perirenal and perigonadal fat were completely dissected out, weighed, frozen, and stored at −80°C until RT-qPCR analyses. The weights of the individual fat depots were used to determine total body fat mass. Liver samples and the brains were also collected, frozen and stored at −80°C. Then, the hypothalamus was dissected out from the base of the brain according the rat brain atlas of Paxinos and Watson (2007).

### Measurement of metabolites, hepatic enzymes, and leptin in plasma

The following plasma metabolites and enzymes were measured: basal glucose, triglycerides, total cholesterol, high-density lipoprotein (HDL), urea, bilirubin, alkaline phosphatase (ALKP), and the hepatic enzymes alanine aminotransferase (ALT), aspartate aminotransferase (AST), and gamma-glutamyl transpeptidase (γGT). These metabolites were analyzed using commercial kits according to the manufacturer's instructions and a Hitachi 737 Automatic Analyzer (Hitachi Ltd, Tokyo, Japan). Very low-density lipoprotein (VLDL) were estimated following the Friedewald equation (Warnick et al., 1990) and low-density lipoprotein (LDL) was determined by the modification of Friedewal equation proposed by Ahmadi et al. (2008): VLDL = TG/5; LDL = [(TChol/1.19)+(TG/1.9)−(HDL/1.1)−38]. The plasma levels of leptin were measured using a commercial rat leptin ELISA kit (Cat. no. RD291001200R; BioVendor, Brno, Czech Republic).

### Measurement of adiposity

Adiposity was estimated by calculating the percentage of abdominal fat weight over total body weight. The sum of the total deposits of perirenal and perigonadal fat determines the amount of abdominal fat.

### RNA isolation and real-time quantitative PCR analysis

We performed real-time qPCR (TaqMan, Life Technologies) as described previously (Decara et al., 2012). Portions (100–300 mg) of liver and PAT, and the whole hypothalamus were homogenized in ice and RNA was extracted using the Trizol® method according to the manufacturer's instruction (Gibco BRL). Reverse transcription was carried out from 1 μg of RNA using the Transcriptor Reverse Transcriptase kit and random hexamer primers (Transcriptor RT, Roche). Real-time qPCR was performed using a CFX96 Touch™ Real-Time PCR Detection System (Bio-Rad). Primers used were obtained based on TaqMan® Gene Expression Assays and the FAM™ dye label format (ThermoFisher; Table S1). We analyzed various housekeeping genes and selected the most suitable according to their homogeneity. Absolute values from each sample were normalized with regard to the housekeeping gene *Actb*. The relative quantification was calculated using the ΔΔCt method and normalized to the control group.

### Statistical analysis

All data are expressed as mean ± S.E.M. Statistical analysis was performed by using SPSS 20.0 for windows (SPSS Inc., Chicago, IL, USA) and GraphPad Prism version 6.0 (GraphPad Software Inc., San Diego, CA, USA). When appropriate, data from body weight, caloric intake and compulsive feeding test over time were analyzed by Student's *t*-test or three-way repeated measures analysis of variance (ANOVA) with time, diet and sex as factors. Data from the feeding response to AM251 were analyzed by two-way ANOVA with treatment and diet as factors. Data from gene expression were analyzed by two-way ANOVA with diet and sex as factors. In all cases, multiple comparisons were assessed by Bonferroni *post-hoc* test. A *P* < 0.05 was considered statistically significant.

## Results

### Effect of prenatal caloric restriction on body weight and caloric intake in dams and their offspring in gestation, lactation, and/or postweaning periods

**Figure 2:**
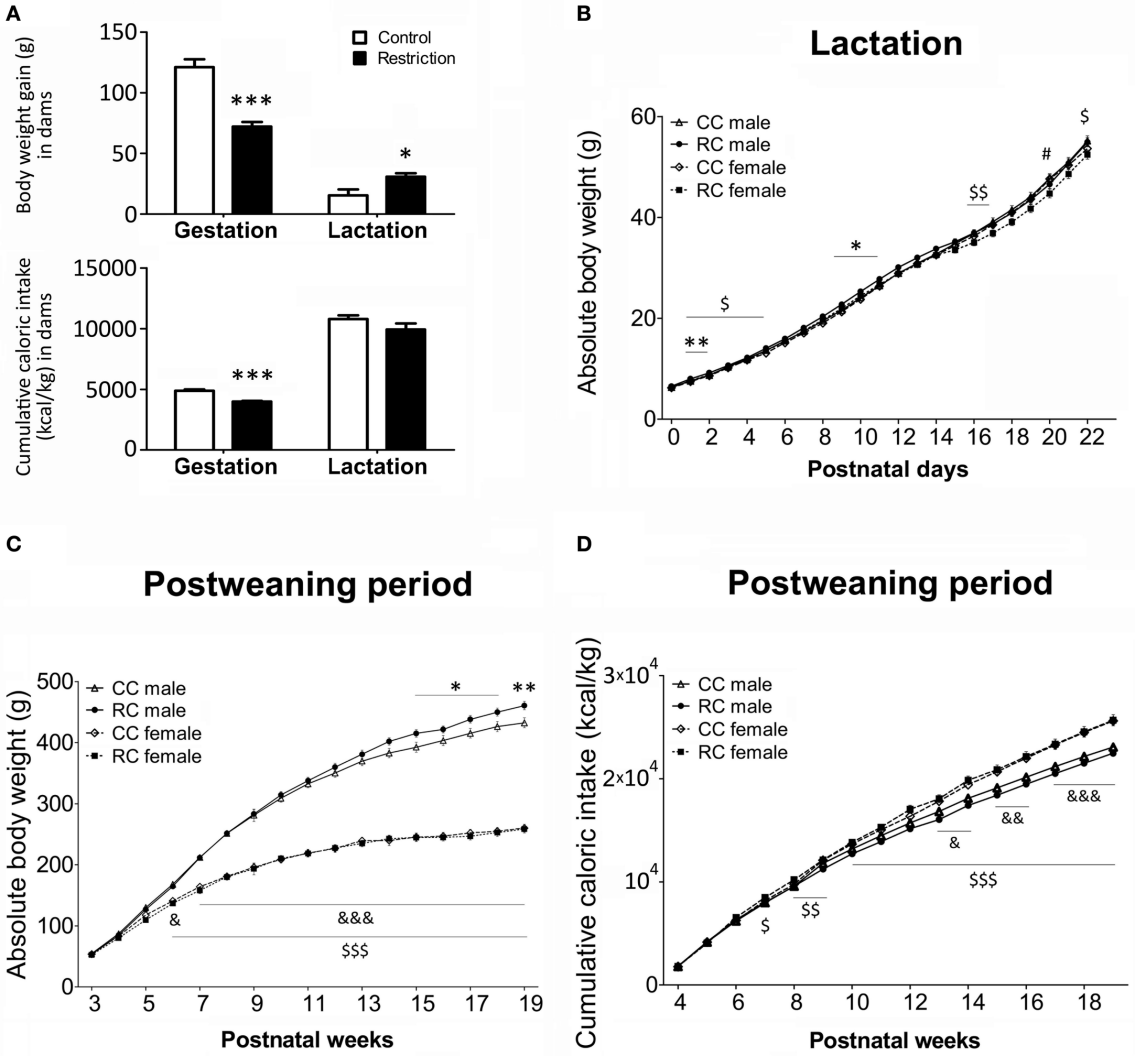
**Body weight (g) and caloric intake (kcal/kg) in dams (A)** and their offspring **(B–D)** in gestation, lactation and/or postweaning periods. Values are expressed as means ± S.E.M. **(A)** Student's *t*-test. ^*/***^P < 0.05/0.001 vs. control dams. **(B–D)** Bonferrroni *post-hoc* test: ^*/**^P < 0.05/0.01 for CC vs. RC males; ^#^*P* < 0.05 for CC vs. RC females; ^&/&&/&&&^*P* < 0.05/0.01/0.001 for CC males vs. females;^$/$$/$$$^*P* < 0.05/0.01/0.001 for RC males vs. females.

Body weight gain and cumulative caloric intake relative to body weight in dams were evaluated after the gestation and lactation periods (Figure 2A). Statistical analysis indicated a significant decrease in body weight gain and caloric intake in the calorie-restricted dams after gestation. In contrast, calorie-restricted dams exhibited an increase in body weight gain after lactation, despite of the lack of effect on caloric intake during this period.

Body weight and cumulative caloric intake relative to body weight in offspring were evaluated during the lactation and/or post-weaning periods (Figures 2B,C). From birth to PND 22 (lactation), three-way repeated measures ANOVA showed a main and significant effect of time on body weight [*F*_(22, 145)_ = 675.53, *P* < 0.001; Figure 2B]. No effects of diet and sex were detected, but interaction between time and diet was significant [*F*_(22, 145)_ = 11.27, *P* < 0.001]. Particularly, Bonferroni analysis indicated that RC male offspring were born normoweight but displayed higher body weight than CC male offspring in several days of the lactation period (PND 1–2 and PND 9–11). We only found a difference between RC and CC female offspring at PND 20. Additionally, RC males exhibited an increase in body weight than RC females in several days of the lactation period (PND 1 and PND 2–5, 16, 17, and 2). However, no differences in body weight were found between CC males and CC females (Figure 2B).

Regarding the body weight during the post-weaning period (Figure 2C), three-way ANOVA detected a main effect of time [*F*_(16, 58)_ = 689.326, *P* < 0.001] and sex [*F*_(1, 73)_ = 436.92, *P* < 0.001]. Interaction between time and diet, time and sex, and time, diet, and sex were also significant [*F*_(16, 58)_ = 1.83, *P* < 0.05; *F*_(16, 58)_ = 72.72, *P* < 0.001; *F*_(16, 58)_ = 2.72, *P* < 0.01, respectively]. Specifically, Bonferroni analysis indicated that RC males also displayed higher body weight than CC males in several weeks of the post-weaning period that comprise the adulthood (postnatal weeks 15–18 and postnatal week 19). However, no differences in body weight between CC and RC females were found. Additionally, both CC and RC males exhibited an increase in body weight compared to the respective CC (postnatal week 7 on) and RC (postnatal week 6 on) females in a time period that comprise most weeks of the adolescence and adulthood (Figure 2C).

Concerning to cumulative caloric intake relative to body weight during the post-weaning period (Figure 2D), we found a main effect of time [*F*_(15, 59)_ = 1712.38, *P* < 0.001] and sex [*F*_(1, 73)_ = 30.20, *P* < 0.001]. Additionally, we detected interaction between time and diet [*F*_(15, 59)_ = 2.19, *P* < 0.05], time, and sex [*F*_(15, 59)_ = 13.28, *P* < 0.001] and time, diet, and sex [*F*_(15, 59)_ = 1.92, *P* < 0.05]. Particularly, Bonferroni analysis indicated that RC males and RC females did not displayed higher caloric intake compared to the respective CC males and CC females. CC females exhibited an increased caloric intake than CC males from the 13th postnatal week (adulthood) onward, meanwhile RC females showed a higher caloric intake than RC males from the 7th postnatal week (adolescence) to adulthood (Figure 2D).

### Long-term effect of prenatal caloric restriction on food preference in adolescent and adult offspring

**Figure 3:**
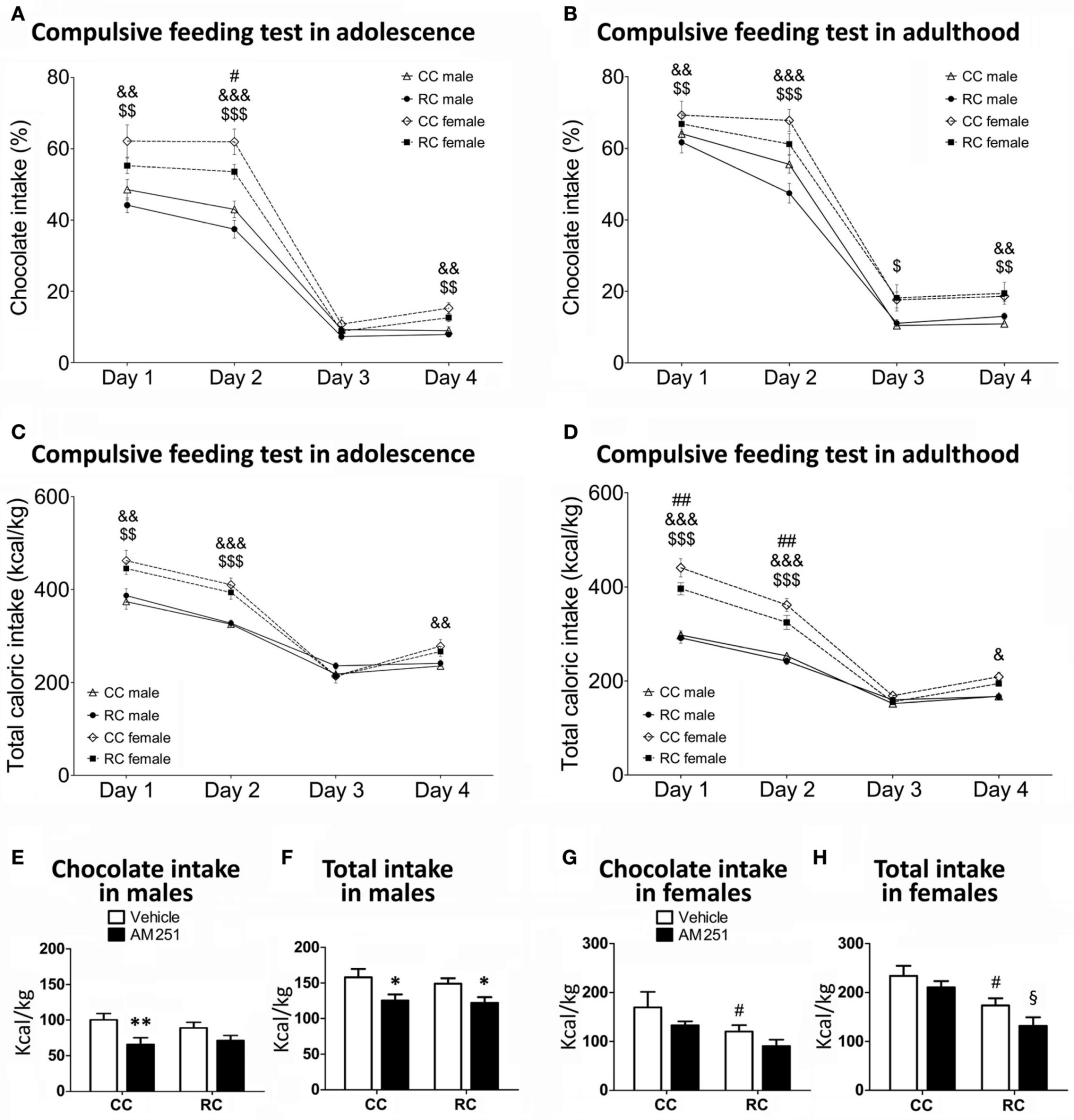
**Compulsive feeding (A–D)** and feeding response to AM25 **(E–H)** for chocolate preference (%) and caloric intake (kcal/kg) in offspring in adolescence and/or adulthood. Values are expressed as means ± S.E.M. Bonferrroni *post-hoc* test: ^*/**^P < 0.05/0.01 vs. respective vehicle males; ^#/##^*P* < 0.05 for CC vs. RC (vehicle) females; ^&/&&/&&&^*P* < 0.05/0.01/0.001 for CC males vs. females;^$/$$/$$$^*P* < 0.05/0.01/0.001 for RC males vs. females;^§^*P* < 0.05 vs. AM251-treated CC females.

Compulsive feeding test (chocolate preference and total caloric intake) was evaluated in adolescent and adult offspring at postnatal weeks 8–9 and 12–13, respectively (Figures 3A–D). Regarding chocolate preference in adolescence (Figure 3A), three-way ANOVA showed a main effect of time [*F*_(3, 65)_ = 474.01, *P* < 0.001], diet [*F*_(1, 67)_ = 7.49, *P* < 0.01], and sex [*F*_(1, 67)_ = 37.81, *P* < 0.001]. We also detected interaction between time and sex [*F*_(3, 65)_ = 13.55, *P* < 0.001]. Bonferroni analysis showed that RC adolescent females exhibited a decrease in chocolate preference at the second day of the test. Additionally, CC and RC adolescent females showed an increase in chocolate preference than the respective CC and RC adolescent males (days 1, 2, and 4; Figure 3A). At adulthood (Figure 3B), we found a main effect of time [*F*_(3, 71)_ = 462.90, *P* < 0.001] and sex [*F*_(1, 73)_ = 13.99, *P* < 0.001] on chocolate preference. We also detected interaction between time and diet [*F*_(3, 71)_ = 3.64; *P* < 0.05], and time and sex [*F*_(3, 71)_ = 2.954, *P* < 0.05]. Bonferrroni analysis indicated that CC and RC adult females displayed higher chocolate preference than the respective CC and RC adult males (days 1, 2, and 4). However, no differences in chocolate preference were found between CC and RC offspring at adulthood (Figure 3B).

Concerning to total caloric intake relative to body weight in adolescence (Figure 3C), we detected a main effect of time [*F*_(3, 65)_ = 272.872; *P* < 0.001], sex [*F*_(1, 67)_ = 25.663; *P* < 0.001], and interaction between time and sex [*F*_(3, 65)_ = 22.908; *P* < 0.001]. Specifically, Bonferroni analysis indicated that CC and RC adolescent females displayed higher caloric intake than the respective CC and RC adolescent males (days 1, 2, and/or 4). No differences in the caloric intake were found between CC and RC offspring at adolescence (Figure 3C). At adulthood (Figure 3D), we observed a main effect of time [*F*_(3, 71)_ = 367.04, *P* < 0.001], diet [*F*_(1, 73)_ = 6.49, *P* < 0.05], and sex [*F*_(1, 73)_ = 124.57, *P* < 0.01]. Interactions between time and sex [*F*_(3, 71)_ = 34.005, *P* < 0.01], and diet and sex [*F*_(1, 73)_ = 4.58, *P* < 0.05] were found. Specifically, Bonferroni analysis indicated that RC adult females exhibited a reduction in caloric intake compared to CC adult females at days 1 and 2 of the test. Moreover, CC and RC adult females displayed higher caloric intake than the respective CC and RC adult males (day 1, 2, and 4; Figure 3D).

### Long-term effect of prenatal caloric restriction on feeding response to AM251 in adult offspring

The feeding behavior to AM251 (chocolate and total caloric intake) was evaluated for 4 h in adult offspring at postnatal weeks 13–14 (Figures 3E–H). Regarding male offspring, two-way ANOVA indicated that there was not prenatal diet effect on chocolate intake and total caloric intake in a treatment-independent manner. However, we detected a main effect of treatment on chocolate intake [*F*_(3, 30)_ = 9.917, *P* < 0.01] and total caloric intake [*F*_(3, 30)_ = 11.189, *P* < 0.01]. Bonferrroni analysis showed that CC males treated with AM251 exhibited a lower chocolate intake than vehicle-treated CC males. This difference was abolished in RC males (Figure 3E). In contrast, both CC and RC males treated with AM251 showed a decrease in the total caloric intake (Figure 3F). Regarding female offspring, two-way ANOVA indicated a main effect of prenatal diet [*F*_(3, 31)_ = 7.517, *P* < 0.05] and an almost significant effect of treatment [*F*_(3, 31)_ = 3.928, *P* = 0.056] on chocolate intake (Figure 3G). Similarly, we detected a main effect of prenatal diet [*F*_(3, 31)_ = 12.458, *P* < 0.01] and an almost significant effect of treatment [*F*_(3, 31)_ = 3.853, *P* = 0.059] on total caloric intake (Figure 3H). Food restriction in dams produced a decrease in caloric intake of female offspring treated with AM251 (Figure 3H).

### Long-term effect of prenatal caloric restriction on plasma levels of leptin, metabolites, alkaline phosphatase, and hepatic transaminases in adult offspring

Two-way ANOVA showed an effect of prenatal diet on the plasma levels of glucose, the lipid profile (triglycerides, HDL, LDL, and VLDL) and urea (Table 1). In addition, a significant effect of sex on the plasma levels of total cholesterol, HDL, ALKP, and AST/ALT ratio was also observed. Interaction between diet and sex was found in the plasma levels of bilirubin and ALT (Table 1), and an almost significant interaction in leptin levels [*F*_(1, 18)_ = 3.349, *P* = 0.084]. Bonferroni analysis indicated that the RC males showed a significant increase in the plasma levels of leptin, triglycerides, VLDL, and bilirubin compared to the plasma of CC males. The RC females presented an increase in the plasma levels of glucose. The plasma levels of LDL were significantly increased in RC males and females. The plasma levels of HDL were significantly decreased in RC males and females. Additionally, RC females showed lower plasma levels of leptin, total cholesterol, LDL, and bilirubin than RC males. The plasma of CC and RC females presented a higher AST/ALT ratio than the plasma of CC and RC males, respectively (Table 1).

**Table 1:** **Plasma levels of leptin, metabolites, alkaline phosphatase, and hepatic transaminases in adult rat offspring at postnatal weeks 21–23^†^**.

	**Male**		**Female**		**Two-way ANOVA**		
	**CC (*n* = 6)**	**RC (*n* = 7)**	**CC (*n* = 5)**	**RC (*n* = 7)**	**Interaction**	**Prenatal diet**	**Sex**
Leptin (ng/mL)	5.82 ± 0.88	12.06 ± 2.13^*^	6.07 ± 2.17	5.97 ± 1.36^$$^	ns	ns	ns
Glucose (mg/dL)	187.33 ± 12.11	207.71 ± 19.27	169.60 ± 15.65	216.00 ± 10.86^#^	ns	*F*_(1, 22)_ = 5.339, *P* = 0.031	ns
Triglycerides (mg/dL)	79.50 ± 3.12	114.13 ± 9.66^*^	82.60 ± 15.96	96.50 ± 11.64	ns	*F*_(1, 22)_ = 5.462, *P* = 0.029	ns
Cholesterol (mg/dL)	39.00 ± 5.49	44.86 ± 2.13	37.20 ± 2.53	31.13 ± 2.56^$$$^	ns	Ns	*F*_(1, 22)_ = 6.112, *P* = 0.022
HDL (mg/dL)	26.67 ± 3.04	4.43 ± 0.81^***^	21.60 ± 2.91	2.12 ± 0.55^###^	ns	*F*_(1, 22)_ = 147.457, *P* < 0.001	*F*_(1, 22)_ = 4.603, *P =* 0.043
LDL (mg/dL)	12.37 ± 2.79	55.67 ± 3.5^***^	17.09 ± 8.37	37.01 ± 7.55^#^,^$^	ns	*F*_(1, 22)_ = 29.887, *P* < 0.001	Ns
VLDL (mg/dL)	15.90 ± 0.62	22.863 ± 1.93^*^	16.52 ± 3.19	19.30 ± 2.33	ns	*F*_(1, 22)_ = 5.462, *P* = 0.029	Ns
Urea (mg/dL)	27.50 ± 2.89	31.25 ± 1.66	30.40 ± 2.02	34.75 ± 2.07	ns	*F*_(1, 22)_ = 4.876, *P* = 0.038	ns
Bilirubin (mg/dL)	0.13 ± 0.02	0.30 ± 0.07^*^	0.20 ± 0.05	0.16 ± 0.02^$^	*F*_(1, 22)_ = 5.733, *P* = 0.026	ns	ns
ALKP (UI)	73.13 ± 3.39	72.14 ± 3.48	63.20 ± 7.79	65.43 ± 2.30	ns	ns	*F*_(1, 23)_ = 4.608, *P* = 0.043
γGT (UI)	10.50 ± 1.01	10.86 ± 0.34	12.20 ± 0.96	11.50 ± 0.93	ns	ns	Ns
AST (UI)	162.50 ± 33.02	138.14 ± 18.33	145.00 ± 20.39	133.14 ± 1.21	ns	ns	Ns
ALT (UI)	55.00 ± 3.96	69.86 ± 6.82	70.40 ± 8.37	56.63 ± 7.27	*F*_(1, 22)_ = 4.697, *P* = 0.041	ns	Ns
AST/ALT	0.39 ± 0.06	0.52 ± 0.03	4.05 ± 0.24^***^	4.84 ± 0.25^$$$^	ns	ns	*F*_(1, 23)_ = 452.066, *P* < 0.001

† *CC, offspring from control-fed dams; RC, offspring from calorie-restricted dams. Values are expressed as means ± S.E.M. Two-way ANOVA and Bonferrroni post-hoc test*:

*/*** *P < 0.05/0.001 vs. CC males*;

#/### *P < 0.05/0.001 vs. CC females*;

$/$$/$$$ *P < 0.05/0.01/0.001 vs. RC males; ns, non-significant*.

### Long-term effect of prenatal caloric restriction on adiposity in adult offspring

We detected a prenatal diet effect on perirenal, perigonadal, and abdominal fat weights, and in the percentage of perirenal and abdominal fat relative to body weight (Table 2). Moreover, we observed a sex effect on perirenal, perigonadal and abdominal fat weights, and in the percentage of perigonadal fat relative to body weight. Interaction between diet and sex was only found in the perirenal fat weight. Bonferrroni analysis indicated that RC male exhibited an increase in the perirenal, perigonadal, and abdominal fat weights, and in the percentage of perirenal fat compared to those of the CC males. No differences in adiposity were observed between CC and RC females (Table 2). CC and RC females showed a lower perirenal, perigonadal, and abdominal fat weights than those of the respective CC and RC males. Moreover, RC females also exhibited a reduction in the percentage of perirenal fat relative to body weight compared to RC males. Based on the main effects on adiposity, the perirenal fat was considered the most suitable adipose tissue for gene expression analysis.

**Table 2:** **Levels of adiposity in perirenal, perigonadal and abdominal fat of rat adult offspring at postnatal weeks 21–23^†^**.

	**Male**		**Female**		**Two-way ANOVA**		
	**CC (*n* = 12)**	**RC (*n* = 15)**	**CC (*n* = 11)**	**RC (*n* = 17)**	**Interaction**	**Prenatal diet**	**Sex**
Perirenal fat (g)	8.90 ± 0.41	12.30 ± 0.64^***^	4.82 ± 0.36^***^	5.73 ± 0.69^$$$^	*F*_(1, 51)_ = 4.214, *P* = 0.045	*F*_(1, 51)_ = 12.542, *P* = 0.001	*F*_(1, 51)_ = 76.281, *P* < 0.001
Perirenal fat/BW (%)	1.97 ± 0.08	2.47 ± 0.12^*^	1.80 ± 0.12	2.06 ± 0.19^$^	ns	*F*_(1, 51)_ = 6.106, *P* = 0.017	ns
Perigonadal fat (g)	9.32 ± 0.61	11.32 ± 0.70^*^	6.14 ± 0.67^**^	7.2 ± 0.49^$$$^	ns	*F*_(1, 51)_ = 4.059, *P* = 0.049	*F*_(1, 51)_ = 34.261, *P* < 0.001
Perigonadal fat/BW (%)	2.06 ± 0.12	2.27 ± 0.13	2.29 ± 0.21	2.66 ± 0.14	ns	Ns	*F*_(1, 51)_ = 4.111, *P* = 0.048
Abdominal fat (g)	18.22 ± 0.94	23.63 ± 1.14^**^	10.96 ± 0.96^***^	12.95 ± 1.11^$$$^	ns	*F*_(1, 51)_ = 11.302, *P* = 0.001	*F*_(1, 51)_ = 66.545, *P* < 0.001
Abdominal fat/BW (%)	4.03 ± 0.18	4.74 ± 0.20	4.15 ± 0.28	4.72 ± 0.30	ns	*F*_(1, 51)_ = 6.163, *P* = 0.016	ns

† *CC, offspring from control-fed dams; RC, offspring from calorie-restricted dams. Values are expressed as means ± S.E.M. Two-way ANOVA and Bonferrroni post-hoc test*:

*/**/*** *P < 0.05/0.01/0.001 vs. CC males*;

$/$$$ *P < 0.05/0.001 vs. RC males; ns, non-significant*.

### Long-term effect of prenatal caloric restriction on the ECS gene expression in hypothalamus, liver, and perirenal adipose tissue of adult offspring

**Figure 4:**
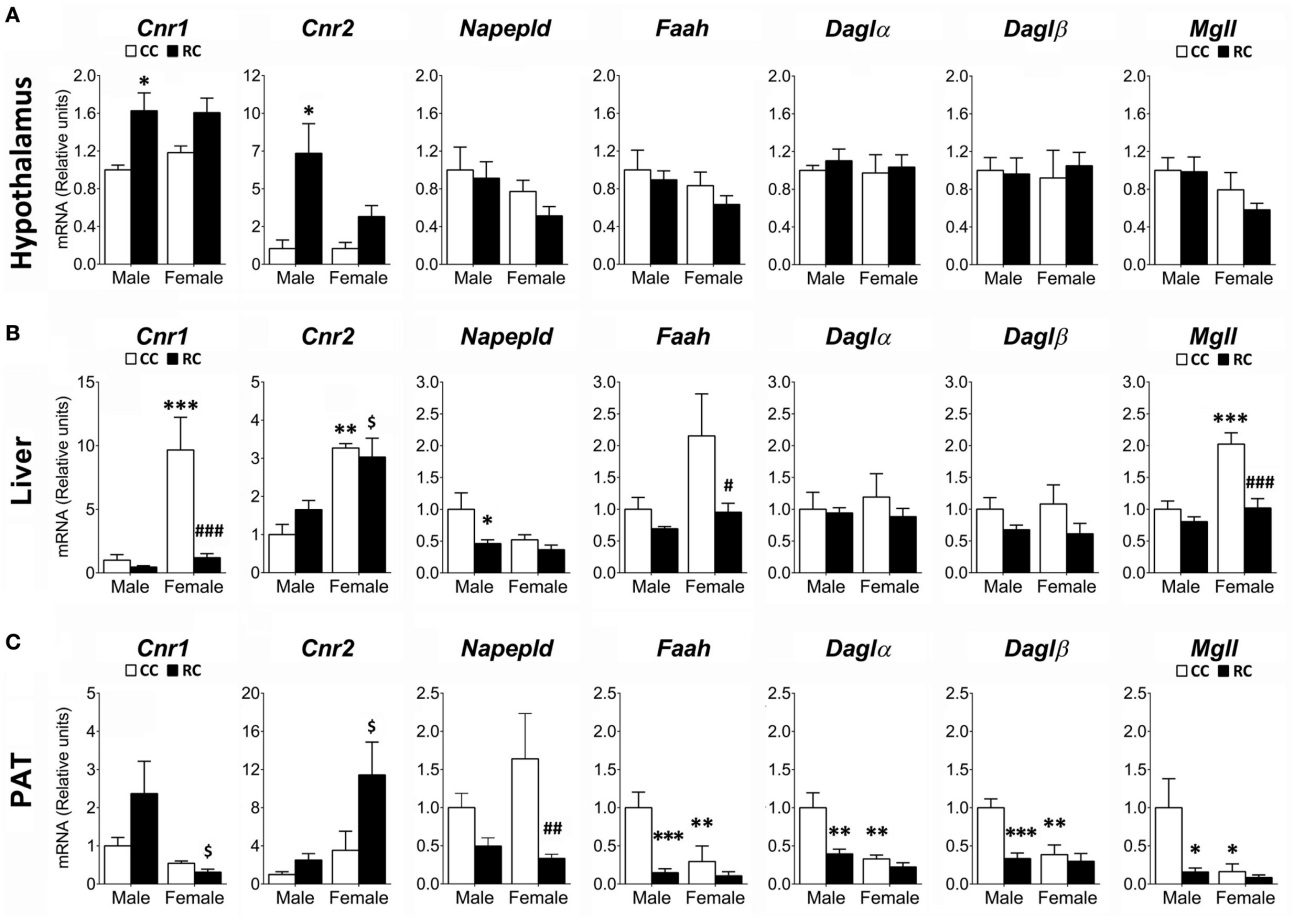
**Effect of prenatal caloric restriction on the gene expression of components of the endocannabinoid system (*Cnr1*, *Cnr2*, *Napepld*, *Faah*, *Dagl*α, *Dagl*β, *Mgll*) in the hypothalamus (A)**, liver **(B)**, and perirenal adipose tissue (PAT, **C**) of male and female offspring at adulthood. Values are expressed as means ± S.E.M. Bonferrroni *post-hoc* test: ^*/**/***^P < 0.05/0.01/0.001 vs. CC males; ^#/##/###^*P* < 0.05/0.01/0.001 vs. CC females; ^$^*P* < 0.05 vs. RC males.

Gene expression of relevant ECS components (*Cnr1, Cnr2, Napepld, Faah, Dagl*α, *Dagl*β, *Mgll*) was evaluated in the hypothalamus, the liver and the PAT of male and female offspring from rat dams which were exposed to an *ad libitum* access of a standard chow (CC) or a 20%-restricted access of the same standard chow (RC) during pregestational (2 weeks) and gestational periods (Figure 4).

Two-way ANOVA showed a main effect of the diet on the gene expression of *Cnr1* in hypothalamus and liver, *Cnr2* in hypothalamus and PAT, *Napepld, Faah, Dagl*β, and *Mgll* in liver and PAT, and *Dagl*α in PAT (see Table S2 for statistical values). We also found a sex effect on the gene expression of *Cnr1, Cnr2*, and *Faah* in liver and PAT, *Mgll* in hypothalamus and PAT, *Napepld* in liver, and *Dagl*α and *Dagl*β in PAT. No interaction between diet and sex was detected in hypothalamus, but it was found in the gene expression of *Cnr1* and *Mgll* in liver, and *Faah, Dagl*α, and *Dagl*β in PAT (Table S2). Bonferroni analysis indicated a significant increase in the gene expression of *Cnr1* and *Cnr2* in the hypoyhalamus of RC male offspring compared to CC ones (Figure 4A). In contrast, the RC males presented lower levels in the gene expression of *Napepld* in liver, and *Faah, Dagl*α, *Dagl*β, and *Mgll* in PAT whereas RC females showed lower levels in the gene expression of *Cnr1, Faah*, and *Mgll* in liver, and *Napepld* in PAT, compared to those of their respective CC offspring (Figures 4B,C). Differences between male and female offspring (CC) were also observed. CC females showed an increase in the gene expression of *Cnr1, Cnr2*, and *Mgll* in liver, but a decrease in the gene expression of *Faah, Dagl*α, *Dagl*β, and *Mgll* in PAT (Figures 4B,C).

### Long-term effect of prenatal caloric restriction on the gene expression of lipid and cholesterol metabolism in liver and perirenal adipose tissue of adult offspring

**Figure 5:**
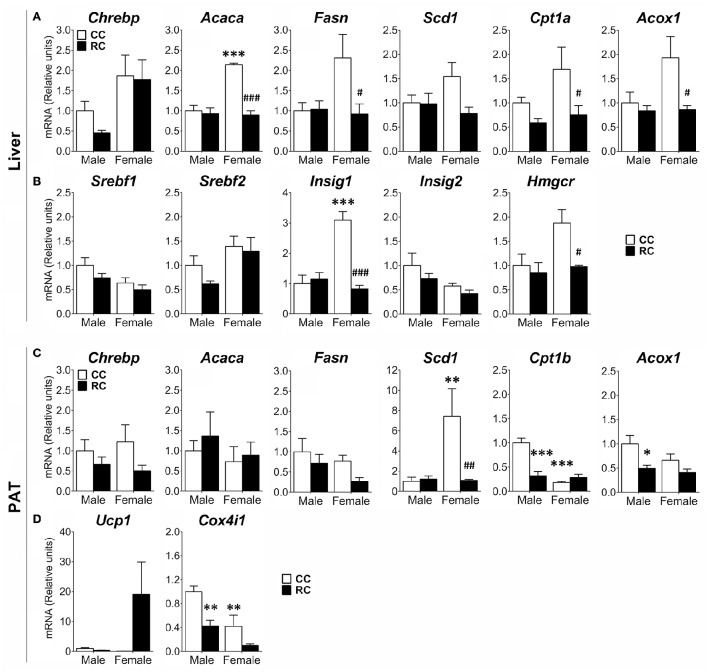
**Effect of prenatal caloric restriction on the gene expression of enzymes and regulators of the lipogenesis (*Chrebp*, *Acaca*, *Fasn*, *Scd1*), fatty-acid β-oxidation (*Cpt1a/b*, *Acox1*), cholesterol metabolism (*Srebf1/2*, *Insig1/2*, *Hmgcr*), and thermogenesis/mitochondria respiration (*Ucp1*, *Cox4i1*) in the liver (A,B)** and/or perirenal adipose tissue (PAT) **(C,D)** of male and female offspring at adulthood. Values are expressed as means ± S.E.M. Bonferrroni *post-hoc* test: ^*/**/***^P < 0.05/0.01/0.001 vs. CC males; ^#/##/###^*P* < 0.05/0.01/0.001 vs. CC females.

Gene expression of relevant metabolic enzymes regulating *de novo* lipogenesis (*Acaca, Fasn, Scd1*) and fatty-acid β-oxidation (*Cpt1a, Cpt1b, Acox1*), and key regulators of lipid and cholesterol metabolism (*Chrebp, Srebp1/2, Insig1/2, Hmgcr*) and mitochondrial respiration (*Ucp1, Cox4i1*) was also evaluated in the liver and the PAT (Figure 5).

Two-way ANOVA showed a main effect of the diet on the gene expression of *Cpt1a, Cpt1b, and Acox1* in liver and PAT, *Acaca, Fasn, Insig1*, and *Hmgcr* in liver, and *Chrebp, Scd1*, and *Cox4i1* in PAT (see Table S3 for statistical values). We also found a sex effect on the gene expression of *Chrebp, Acaca, Acox1, Srebf1/2, Insig1/2*, and *Hmgcr* in liver, and *Scd1, Cpt1b*, and *Cox4i1* in PAT. Interaction between diet and sex was detected in the gene expression of *Acaca, Fasn, Acox1*, and *Insig1* in liver, and *Scd1* and *Cpt1b* in PAT (Table S3). Bonferroni analysis indicated that RC females presented a significant decrease in the gene expression of *Acaca, Fasn, Cpt1a, Acox1, Insig1*, and *Hmgcr* in liver, and *Scd1* in PAT compared to that of CC ones (Figures 5A–C). RC male offspring showed a decrease in the gene expression of *Cpt1b, Acox1*, and *Cox4i1* in PAT (Figures 5C,D). Differences between male and female offspring (CC) were also observed. CC females showed an increase in the gene expression of *Acaca* and *Insig1* in liver, and *Scd1* in PAT, compared to CC males (Figures 5A–C). In contrast, CC females exhibited a decrease in the gene expression of *Cpt1b* and *Cox4i1* (Figures 5C,D).

### Long-term effect of prenatal calorict restriction on the gene expression of *Ppar*α and *Ppar*γ in liver and perirenal adipose tissue of adult offspring

**Figure 6:**
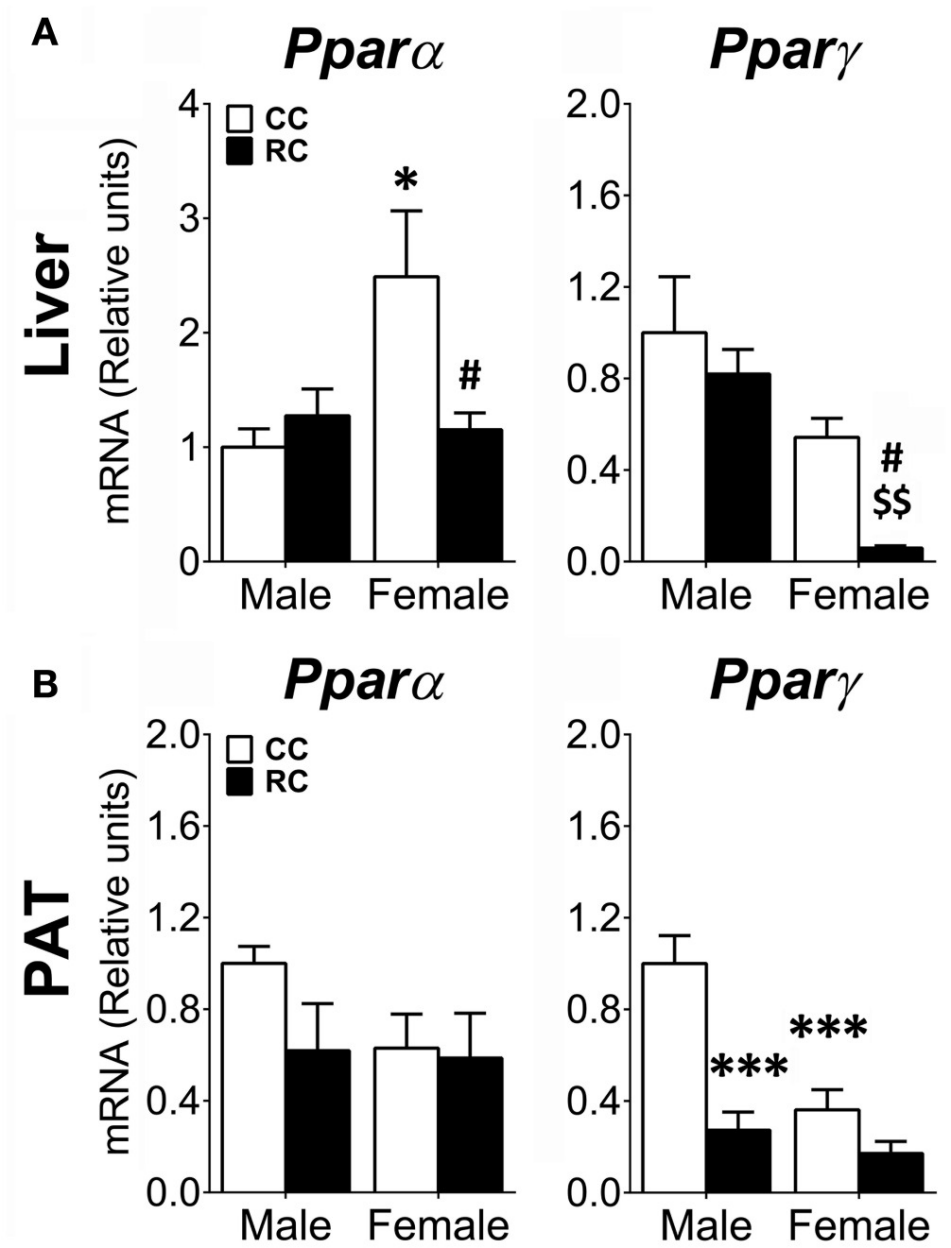
**Effect of prenatal caloric restriction on the gene expression of the lipid flux and storage regulators *Ppar*α and *Ppar*γ in the liver (A)** and perirenal adipose tissue (PAT) **(B)** of male and female offspring at adulthood. Values are expressed as means ± S.E.M. Bonferrroni *post-hoc* test: ^*/***^P < 0.05/0.01/0.001 vs. CC males; ^#^*P* < 0.05 vs. CC females; ^$$^*P* < 0.01 vs. RC males.

Gene expression of relevant transcription factors (*Ppar*α, *Ppar*γ) regulating the expression of genes implicated in lipid and carbohydrate metabolism was also evaluated in the liver and the PAT (Figure 6).

Two-way ANOVA showed effects of diet and sex on the gene expression of *Ppar*γ in liver and PAT (see Table S4 for statistical values). Additionally, a main effect of sex was also detected on the gene expression of *Ppar*α in liver. Interaction between diet and sex was found in the liver *Ppar*α gene expression and the PAT *Ppar*γ gene expression (Table S4). Bonferroni analysis indicated that while RC female offspring showed a decrease in the gene expression of *Ppar*α and *Ppar*γ in liver (Figure 6A), RC males only presented a significant decrease in the gene expression of *Pparg* in PAT (Figure 6B), compared to CC ones. Regarding the differences between male and female offspring, CC females showed an increase in the gene expression of *Ppar*α in liver, but a decrease in the gene expression of *Ppar*γ in PAT (Figures 6A,B).

## Discussion

This study demonstrates that the maternal exposure to a moderate (20%) caloric restriction (undernutrition) during preconceptional (2 weeks) and gestational (up 2 days before birth) periods produced long-term effects on body weight, feeding behavior and gene expression of key components of the ECS (receptors and enzymes) in a sex-dependent manner. These effects extended to lipid and cholesterol metabolic pathways, suggesting the existence of a complex pattern alterations derived of adaptation to maternal malnutrition.

### A moderate maternal caloric restriction produces sex-dimorphic effects in feeding and body weight gain only after weaning

The dams exposed to undernutrition during prenatal stages exhibited a reduction in their body weight gain and caloric intake during gestation that contrasts with the increase in their body weight during lactation with normal nutrition. Moreover, their offspring displayed normoweight at birth and during lactation in a sex and diet-independent manner. This finding is in agreement with previous studies in humans, which demonstrated that offspring exposed to famine or hyperemesis gravidarum in the first part of pregnancy did not show lower birth weight (Roseboom et al., 2006; Grooten et al., 2015). Similarly, animal studies documented normoweight at birth when the early pregnancy was conducted under caloric restriction (Cleal et al., 2007; Palou et al., 2010; Poore et al., 2010). In contrast, a lower weight at birth has been described in several studies when the calorie-restricted diet was prolonged to birth (Desai et al., 2005; Lukaszewski et al., 2011). An explanation that emerges from these studies is that the putative fetal underweight caused by maternal undernutrition may be recovered in few days of normal nutrition, as was previously suggested (Cleal et al., 2007; Ramírez-López et al., 2016). Returning to our data, weight recovery in offspring may occur as a result of an accelerated catch-up growth before birth caused by an *ad libitum* maternal feeding.

The differential response to the maternal undernutrition in energy balance is sexual dimorphism dependent during the post-weaning periods. The results indicated that CC male rats showed an increase in body weight and abdominal fat mass that contrasted with a decrease in caloric intake and compulsive feeding during the adolescence and adulthood compared to CC female rats. As we will discuss below, these sex-differences extend to metabolic pathways under the control of the endogenous cannabinoid system, as previously suggested (Wagner, 2016). Thus, the effects of the maternal caloric restriction on the energy balance of male and female offspring should be interpreted based on their sex-dependent basal metabolism.

The adult male offspring from calorie-restricted dams exhibited an increase in body weight and a higher restriction in total caloric intake after the administration of AM251, a CB1 receptor inverse agonist with anorexigenic and anti-obesity properties. The increase in body weight of RC male offspring is directly associated with an increase in abdominal fat and, particularly, a higher percentage of perirenal adiposity relative to body weight. The elevated fat mass and body weight justify the higher levels of triglycerides, LDL, VLDL, bilirubin, and the adipocytes-specific satiety hormone leptin in plasma. In contrast, the female offspring from calorie-restricted dams did not show changes in body weight and adiposity, but exhibited higher levels of LDL and glucose in plasma, and a reduction in chocolate preference at adolescence and total caloric intake at adulthood. The lower compulsive feeding in RC females can be associated with the hyperglycemia via a hypothalamic impairment of glucose-sensing to control hunger and satiety (Routh et al., 2014).

Previous researches have demonstrated metabolic alterations after undernutrition in a sex-dependent manner. Increased adiposity, lipid profile, glycemia, and leptinemia in male offspring have been reported after the exposure to a moderate undernutrition at different pregnancy periods (Desai et al., 2007a; Palou et al., 2010). Concerning the potential explanations of the differential phenotypic features between males and females, the leptin sensibility can play an important role. Indeed, the higher leptin levels in plasma without reduction in food intake suggest leptin resistance and, therefore, an altered leptin signaling (Friedman and Halaas, 1998). A putative decrease in hypothalamic sensitivity to leptin may result in an inability to detect satiety despite the fat stores, which finally develop to obesity (Sasaki, 2015). The higher leptin sensitivity described in females than in males has been associated with gonadal hormones (Clegg et al., 2003, 2006) and may explain in part the higher levels of leptin found in the plasma of male offspring. Furthermore, the placenta responses to maternal diet in a sex-specific manner (Tarrade et al., 2015), suggesting that the sexual dimorphism in adiposity-relevant parameters could be present at very early stages.

### Effects of moderate maternal caloric restriction on feeding behavior

This differential sex effect on body weight after maternal caloric restriction was in agreement with data from previous studies (Palou et al., 2010). The obvious explanations to the higher body weight found in RC males after weaning could be as a consequence of an increased food intake, decreased energy expenditure or higher metabolic efficiency. Concerning hyperphagia, several studies have reported this feeding alteration after the exposure to undernutrition in critical windows of development (Vickers et al., 2000; Desai et al., 2005; Breton et al., 2009; Palou et al., 2010; Lukaszewski et al., 2011; Manuel-Apolinar et al., 2014), so it has been proposed as an important contributor of the excessive body weight. Intriguingly, our data indicated that either male or female offspring from calorie-restricted dams did not display higher caloric intake after weaning, despite of the fact that the males exhibited greater body weight at adulthood. Indeed, discrepancies were also found in the literature. For instance, it has been also documented excessive body weight in absence of higher food intake (Yura et al., 2005; Sebert et al., 2009; Theys et al., 2011). Evidence in some studies pointed out that hyperphagia could be a transitory effect that disappears when the underweighted animals after maternal undernutrition were able to reach the weight as controls (Vickers et al., 2000; Lukaszewski et al., 2011). So, hyperphagia could be a compensatory effect after prolonged underweight. In this context, it is plausible in our experimental model that RC offspring did not showed underweight and, therefore, hyperphagia in any stage. It is important to note that when caloric intake is adjusted to body weight the effect of maternal caloric restriction on appetite seems more discrete (Lagisz et al., 2014). One possibility that emerges from our data is that male offspring can increase their body weight by either a reduction of energy expenditure or higher metabolism efficiency. This fact might be a consequence of the inadequate fetal programming of metabolic systems involved in energy homeostasis (Hales and Barker, 2001; Vaag et al., 2012). The analysis of the cannabinoid-regulated metabolism, such as the expression of key enzymes, receptors and regulators of the lipid and cholesterol metabolism, could provide some clues to what may be happening.

In order to further investigate the effect of maternal caloric restriction on feeding behavior we performed a compulsive feeding test in the adult offspring. The effect observed in the RC offspring were contrary to those from previous studies, which described either, an increased preference for higher caloric diets after exposure to caloric restriction during pregnancy (Lussana et al., 2008; Palou et al., 2010) or hyperphagia in animals fed with a high-fat diet (Lukaszewski et al., 2011). Different factors can partially explain the discrepancies found in food preferences. Regarding the time exposure to diet, Palou et al. (2010) described a higher preference for a fat-rich liquid diet than a carbohydrate-rich liquid diet in male offspring fed for 1 h after an adaptation period of 8 days (habituation to diet). However, our data indicated a lower preference for a novel highly-palatable food (chocolate mixture) than the familiar standard diet in RC females when they were exposed for 4 days. The lower acceptance to novel foods has been found in animals with protein deficiency from early gestational stages (Peregoy et al., 1972; Pettus et al., 1974) and could be associated with abnormal behaviors in adulthood related to anxiety (Peleg-Raibstein et al., 2012). Regarding the timing of restriction, protein deficiency during early pregnancy induces a less preference to a high-fat diet in the female offspring (Bellinger and Langley-Evans, 2005), whereas a high preference was obtained when restriction was applied through other gestational periods (Bellinger et al., 2004; Bellinger and Langley-Evans, 2005). According to our results, a maternal caloric restriction prior to mating could be critical for the less compulsive feeding observed in female offspring.

The tendency to lower chocolate preference suggests an increased threshold of the palatability. Interestingly, the ECS is implicated in the intake of palatable food (DiPatrizio and Simansky, 2008) and the perception of sweet taste (Niki et al., 2015). The CB1 receptor inverse agonist AM251 was able to decrease the motivation to obtain highly palatable food in a free choice test (Mathes et al., 2008; Droste et al., 2010; Deshmukh and Sharma, 2012). According to this, AM251 was specifically able to reduce chocolate intake in CC male offspring, but not in CC females or RC males. Interestingly, caloric restriction induced a higher reduction in total intake in the female offspring treated with AM251. This subtle difference may indicate some alterations in the central rewarding functions of the ECS, as was previously described in animals with reduced preference for highly palatable foods (Brand et al., 2012).

### Impact of maternal caloric restriction on the expression of the endogenous cannabinoid system in organs related to energy expenditure and metabolism

The sex-dependent effects of maternal caloric restriction on feeding behavior and metabolic profile in offspring can be a result of a differential re-programming in energy metabolism. Moreover, long-lasting changes in the gene expression of components of the ECS, which modulates appetite and energy homeostasis, and other key enzymes and regulators of lipid and cholesterol metabolism, were found in metabolically relevant tissues (liver and PAT) of adult offspring in a sex-dependent manner. The adult male offspring from calorie-restricted dams specifically exhibited an increased gene expression of the cannabinoid receptors *Cnr1* and *Cnr2* in the hypothalamus, and showed a decreased gene expression of the eCBs-metabolizing enzymes *Napepld* in liver, and *Faah, Dagl*α, *Dagl*β, and *Mgll* in PAT. These results likely indicate that the up-expression of the cannabinoid receptors in the hypothalamus at adulthood may result from a lower long-term cannabinoid activity that, in turn, could be produced by lower levels of eCBs and N-acylethanolamines (NAEs) in the hypothalamus of male offspring as was previous described at birth (Ramírez-López et al., 2016). This interpretation also agrees with the down-regulation of the endocannabinoid metabolism in peripheral tissues. However, the adult female offspring from calorie-restricted dams showed a decreased gene expression of *Cnr1* and the eCBs-degrading enzymes *Faah* and *Mgll* in liver as well as the NAEs-synthesizing enzyme *Napepld* in PAT. The prominent down-regulation of the eCBs-degrading enzymes in the liver of RC females may result in a different endocannabinoid signaling than that of RC males. Higher endocannabinoid levels as a result of a lower expression of the eCBs-degrading enzymes FAAH and MAGL in liver can be associated with alterations in the lipid and cholesterol metabolism (Dinh et al., 2002; Osei-Hyiaman et al., 2005; Di Marzo and MacCarrone, 2008), due to the fact that both eCBs anandamide (AEA) and 2-AG can inhibit the gene expression of apolipoprotein A1, the primary protein component of HDL, through the activation of CB1 receptors in hepatocytes (Haas et al., 2012). Despite of these alterations, in general, the female phenotype seems less severely affected after the exposure to maternal undernutrition in early development. Any explanation for these dimorphisms should be supported by the elevated gene expression of *Cnr2* in the liver and PAT of the RC female offspring compared to those of the RC males. Interestingly, the CB2 receptor activation was associated with anti-obesity effects (Verty et al., 2015) and a decreased risk for cardiovascular diseases. For instance, the gene deletion of the 2-AG-degrading enzyme *Mgll* has been linked to attenuation of diet-induced insulin resistance and improvement of atherosclerosis via CB2 activation (Taschler et al., 2011; Vujic et al., 2016). Taken in account the lower impact of the exposure to undernutrition observed in females in particular, and the lower risk of cardiovascular diseases described in females in general (Blenck et al., 2016), we can speculate that the CB2 receptor up-regulation may play a protective role in adverse metabolic conditions.

The higher gene expression of *Cnr1* in the hypothalamus, which was subtly more evident in male than female offspring from caloric-restricted dams, was not so clear in previous studies (Matias et al., 2003). Matias et al. (2003) demonstrated a decrease in the hypothalamic levels of AEA (confirmed by Ramírez-López et al., 2016) and an absence of significant changes in the gene expression of *Cnr1* and *Faah* in the hypothalamus of weaned pups after a maternal undernutrition throughout the last part of pregnancy and/or lactation. A possible explanation of this discrepancy could be related to the critical consequences of the maternal undernutrition at preconceptional periods. The up-expression of *Cnr1* in the hypothalamus has been associated with the leptin resistance in obesity (Thanos et al., 2008; Cardinal et al., 2012). This fact agrees with the increased body weight and the higher plasma levels of leptin in RC male offspring despite of the absence of hyperphagia or alterations in hypothalamic endocannabinoid machinery. It was described that long-term undernutrition from early stages lead to higher gene expression of *Cnr1* in several brain regions of the leptin-deficient obese Zucker rats (Thanos et al., 2008). These findings indicate that leptin action and, therefore, the normal function of leptin receptors reduces the endocannabinoid tone and CB1 receptor activity (Di Marzo et al., 2001). Regarding the absence of hyperphagia, it is important to note that the food intake has been associated with the cannabinoid activation in specific hypothalamic areas (Soria-Gomez et al., 2014). However, previous studies suggested that specific CB1 receptor deletion in the hypothalamus was able to decrease body weight and increase energy expenditure, but was not accompanied with changes in food intake (Cardinal et al., 2012).

The hypothalamus is able to cross-talk to other metabolically relevant tissues such as liver and adipose tissue through sympathetic circuits and circulating signals (leptin, insulin, CCK, neurotrophins, IL-6) by the homeostatic regulation of the endocannabinoid signaling system (Das, 2010; Maccarrone et al., 2014; Seoane-Collazo et al., 2015). Therefore, an alteration in the hypothalamic levels of endocannabinoids in newborn animals, as was previously described by Ramírez-López et al. (2016), could impair the sympathetic neurotransmission and hormones involved in energy metabolism (Di Marzo et al., 2001; Keimpema et al., 2013, 2014; Cristino et al., 2014). Thus, the down-expression of the eCBs-degrading enzymes *Faah* and *Mgll* in the PAT of RC male offspring is consistent with an obese phenotype, hyperleptinemia, adiposity, and alterations in lipid metabolism in absence of increased food intake, as was previously described in animals with deficiency of FAAH and MAGL (Tourino et al., 2010; Geurts et al., 2015). In contrast, the decreased gene expression of the 2-AG-synthesizing enzymes *Dagl*α and *Dagl*β is opposed to what was previously found in the adipose tissue of obese subjects (Engeli et al., 2014). Therefore, the down-regulation of the endocannabinoid machinery in the adipose tissue of male offspring at adulthood may result in a putative compensatory effect in order to respond to an unbalanced endocannabinoid tone originated at birth after maternal undernutrition.

### Maternal caloric restriction induces sex-dimorphic alterations in the expression of genes involved in lipid metabolism

Sexual dimorphism in offspring after maternal caloric restriction was also observed in the gene expression of key enzymes and regulators of the lipid and cholesterol metabolism that include elements of the fatty-acid β-oxidation. The PAT of adult male offspring from calorie-restricted dams specifically exhibited a decrease in the gene expression of the β-oxidation enzymes *Cpt1b* and *Acox1*, the mitochondrial respiratory chain subunit *Cox4i1* and the lipid storage-stimulating transcription factor *Ppar*γ. The down-expression of all these elements in PAT indicates lower energy expenditure in RC males, which agrees with the increased perirenal adiposity and the higher levels of triglycerides in plasma. These features were also linked to an impairment of the browning process of the white adipose tissue (Geurts et al., 2015). Despite we found a decrease in the gene expression of *Ppar*γ, opposite results after the exposure to undernutrition (Bispham et al., 2005) and similar results after malnutrition during early development have been documented (Ahmad et al., 2013; Reynolds et al., 2014). Interestingly, the decreased gene expression of *Ppar*γ has been linked to inflammatory processes associated with obesity and insulin resistance (Odegaard et al., 2007; Reynolds et al., 2014), an issue that enhances the importance of the inflammatory responses in the developmental programming.

The liver of adult female offspring from calorie-restricted dams specifically showed a decrease in the gene expression of *Ppar*α and *Ppar*γ. As these nuclear receptors has been also linked to the activation of fatty-acid β-oxidation and insulin sensitivity (Auwerx, 1999; Minnich et al., 2001), their decreased levels in RC females might be associated to alterations in lipid and glucose metabolism (Liu et al., 2015). The liver of RC females also showed a decrease in the gene expression of the *de novo* lipogenic enzyme *Acaca* and *Fasn*, which constitute rate-limit steps in the fatty-acid synthesis, and the liver-specific cholesterol biosynthesis regulators *Insig1* and *Hmgcr*. The down-expression of these elements suggests a reduction of fatty-acid and cholesterol biosynthesis in the liver of RC females, which could partially explain the altered levels of the lipid carriers high-density, low-density and very low-density lipoproteins (HDL, LDL, and HLDL) in plasma. The HDL was 10-fold reduced in the plasma of RC females, which indicates a significant reduction of the outlet rate of fat molecules (cholesterol, phospholipids and triglycerides) from cells. In contrast, the LDL was two-fold increased in the plasma of RC females, which is strongly associated with artherosclerosis within the artery wall and poses a risk for cardiovascular disease. Similarly to human cohort studies, our results emphasize the importance of early life programming as the exposure to undernutrition during early development increases the risk from suffering features of metabolic syndrome and cardiovascular disease later in life (Desai et al., 2005, 2007b; Jimenez-Chillaron et al., 2005; Yura et al., 2005; Breton et al., 2009; Palou et al., 2010; Suzuki et al., 2010; Lukaszewski et al., 2011; Theys et al., 2011).

### Sexual dimorphisms: an open question

A major finding of this study is the appearance of sexual dimorphisms on the effects of maternal caloric restriction in the adult offspring. Differences extend not only to metabolic parameters and gene expression but also to behavioral responses. A major issue in sex differences is the need for control the estrous phase on which the evaluations were performed in order to clarify the impact of sex hormones variations in the female. Since the number of animals needed to control the three phases of the estrous cycle, including morning to afternoon time variations, was too high to be affordable, we had to use randomly cycling female animals sacrificed only during the morning time. This limitation has to be considered in the interpretation of the data and further research will be addressed to evaluate the reasons for the sex-dimorphic alterations. However, it is important to note that in the female rat, sex steroids regulate both the expression and function of the endogenous cannabinoid system, so a potential explanation will also involve the impact of malnutrition on this important modulatory system, as it was described previously (Bonnin et al., 1993; Rodríguez de Fonseca et al., 1994).

## Conclusion

As conclusion, our results demonstrated that the exposure to a moderate maternal restricted diet from the preconceptional period predisposed for the development of features related to the metabolic syndrome, affected subtly the feeding behavior and altered the gene expression of relevant regulators of the lipid and cholesterol metabolism, including the endocannabinoid signaling system, in metabolically-relevant tissues (hypothalamus, liver and PAT) of adult offspring in a sex specific-manner. Moreover, the outcomes of the present study enhance the importance of the periconceptional period as well as the role of maternal diet in the early life programming, due to the fact that the maternal undernutrition was implemented before mating and offspring were raised in an *ad libitum* standard feeding. Importantly, this evidence supports the idea that the contribution of the endocannabinoid system in the early life programming could be critical. Our findings represent an important key point to understand the complexity of the fetal programming process and might be particularly useful in the searching of efficient therapies against a malprogramming. Further studies should be performed to clarify the role of the endocannabinoid system and other homeostatic mechanisms implicated in the process of nutritional programming.

## Author contributions

FR, JS, and RG conceived the study and got financial support. MTRL, MV, RNB, and FA did the animal experiments and sampling. RA, JD, PR, and JS did both, the biochemical and statistical analysis. MTRL, JS, and FR wrote the first draft of the manuscript that was revised by all authors.

## Funding

This work was supported by the Instituto de Salud Carlos III, Ministerio de Economía y Competitividad con-founded by UE-ERDF program (CP12/03109 and PI16/01374 to JS, PI12/02261 to FR, and PSI-2012-35388 to RG), Red de Trastornos Adictivos (RD12/0028/0001 to FR), CIBERobn, Consejería de Economía, Innovación y Ciencia, Junta de Andalucía, UE/ERDF (PI45403, CTS-8221, CTS-433 to FR), Consejería de Salud, Junta de Andalucía, UE/ERDF (SAS111224 to JS and FR. MTRL has been funded by a FPU predoctoral fellowship of the Spanish Ministerio de Educación, Cultura y Deporte (AP-2009-0225); JS holds a “Miguel Servet” research contract from the National System of Health, ISCIII (grant number CP12/03109).

### Conflict of interest statement

The authors declare that the research was conducted in the absence of any commercial or financial relationships that could be construed as a potential conflict of interest.
