# Supplementary material for: Long-Term Effects of Prenatal Exposure to Undernutrition on Cannabinoid Receptor-Related Behaviors: Sex and Tissue-Specific Alterations in the mRNA Expression of Cannabinoid Receptors and Lipid Metabolic Regulators
Source: Front Behav Neurosci. 2016 Dec 27;10:241. doi: 10.3389/fnbeh.2016.00241 (PMC5187359; doi:10.3389/fnbeh.2016.00241)
Supplement: Supplementary file 1 [file Table1.PDF]

Table S1. Primer references for TaqMan® Gene Expression Assays (ThermoFisher)

Gene description	Assay ID	Nº accession GenBank	Amplicon length
Actb (beta actin)	Rn00667869_m1	NM_031144.2	91
Gapdh (glyceraldehyde 3-phosphate dehydrogenase)	Rn01775763_g1	NC_005103.4	174
Cnr1 (cannabinoid receptor type 1)	Rn02758689_s1	NM_012784.4	92
Cnr2 (cannabinoid receptor type 2)	Rn03993699_s1	NM_001164142.1	102
Napepld (N-acyl phosphatidyl ethanolamine phospholipase D)	Rn01786262_m1	NM_199381.1	71
Faah (fatty acid amide hydrolase)	Rn00577086_m1	NM_024132.3	63
Daglα (diacylglycerol lipase, alpha)	Rn01454304_m1	NM_001005886.1	67
Daglβ (diacylglycerol lipase, beta)	Rn01453770_m1	NM_001107120.1	57
Mgll (monoglyceride lipase)	Rn00593297_m1	NM_138502.2	78
Chrebp (carbohydrate-responsive element-binding protein, *Mlxipl*)	Rn00591943_m1	NM_133552.1	84
Acaca (acetyl-CoA carboxylase alpha*)*	Rn00573474_m1	NM_022193.1	60
Fasn (fatty acid synthase)	Rn01463550_m1	NM_017332.1	148
Scd1 (stearoyl-Coenzyme A desaturase 1)	Rn00594894_g1	NM_139192.2	86
Cpt1a (carnitine palmitoyltransferase 1a, liver)	Rn00580702_m1	NM_031559.2	64
Cpt1b (carnitine palmitoyltransferase 1b, muscle)	Rn00682395_m1	NC_005106.4	83
Acox1 (acyl-CoA oxidase 1, palmitoyl)	Rn01460628_m1	NM_017340.2	63
Pparα (peroxisome proliferator activated receptor alpha*)*	Rn00566193_m1	NM_013196.1	98
Pparγ (peroxisome proliferator activated receptor gamma)	Rn00440945_m1	NC_005103.4	105
Srebp1 (sterol regulatory element binding transcription factor 1)	Rn01495769_m1	XM_213329.5	79
Srebp2 (sterol regulatory element binding transcription factor 2)	Rn01502638_m1	NM_001033694.1	61
Insig1 (insulin induced gene 1)	Rn00574380_m1	NM_022392.1	68
Insig2 (insulin induced gene 2)	Rn00710111_m1	NM_178091.4	89
Hmgcr (3-hydroxy-3-methylglutaryl-CoA reductase)	Rn00565598_m1	NM_013134.2	71
Ucp1 (uncoupling protein 1)	Rn00562126_m1	NM_001033694.1	69
Cox4i1 (cytochrome c oxidase subunit 4 isoform 1)	Rn00665001_g1	NC_005118.4	72

Table S2. Interaction and effects of prenatal diet and sex on the gene expression of relevant ECS components (see Figure 4)†

Hypothalamus	Interaction	Prenatal diet	Sex
Cnr1	ns	F(1,23)=11.82	ns
Cnr2	ns	F(1,23)=10.23	ns
Napepld	ns	ns	ns
Faah	ns	ns	ns
Dagla	ns	ns	ns
Daglb	ns	ns	ns
Mgll	ns	ns	F(1,23)=4.87
**Liver**	**Interaction**	**Prenatal diet**	**Sex**
Cnr1	F(1,22)=15.32	F(1,22)=19.73	F(1,22)=21.46
Cnr2	ns	ns	F(1,22)=28.77
Napepld	ns	F(1,21)=8.02	F(1,21)=5.53
Faah	ns	F(1,22)=7.40	F(1,22)=6.51
Dagla	ns	ns	ns
Daglb	ns	F(1,21)=5.05	ns
Mgll	F(1,22)=9.50	F(1,22)=20.88	(1,22)=22.23
**PAT**	**Interaction**	**Prenatal diet**	**Sex**
Cnr1	ns	ns	F(1,23)=5.62
Cnr2	ns	F(1,22)=5.04	F(1,22)=7.46
Napepld	ns	F(1,23)=13.67	ns
Faah	F(1,23)=6.92	F(1,23)=16.84	F(1,23)=8.69
Dagla	F(1,23)=5.87	F(1,23)=11.90	F(1,23)=16.86
Daglb	F(1,18)=7.73	F(1,18)=13.07	F(1,18)=9.76
Mgll	ns	F(1,22)=6.20	F(1,22)=6.06

†Two-way ANOVA

Table S3. Interaction and effects of prenatal diet and sex on the gene expression of relevant components and regulators of lipid and cholesterol metabolism (see Figure 5)[†]

**Liver**	**Interaction**	**Prenatal diet**	**Sex**
Chrebp	ns	ns	F(1,21)=8.96
Acaca	F(1,22)=22.48	F(1,22)=27.74	F(1,22)=19.50
Fasn	F(1,22)=5.35	F(1,22)=4.84	ns
Scd1	ns	ns	ns
Cpt1a	ns	F(1,21)=9.10	ns
Acox1	F(1,22)=4.58	F(1,22)=8.48	F(1,22)=5.06
Srebf1	ns	ns	F(1,22)=6.91
Srebf2	ns	ns	F(1,22)=6.90
Insig1	F(1,22)=28.23	F(1,22)=21.77	F(1,22)=15.08
Insig2	ns	ns	F(1,22)=6.24
Hmgcr	ns	F(1,21)=6.71	F(1,21)=6.19
**PAT**	**Interaction**	**Prenatal diet**	**Sex**
Chrebp	ns	F(1,23)=4.81	ns
Acaca	ns	ns	ns
Fasn	ns	ns	ns
Scd1	F(1,23)=10.03	F(1,23)=9.21	F(1,23)=8.77
Cpt1b	F(1,23)=24.07	F(1,23)=13.12	F(1,23)=28.66
Acox1	ns	F(1,23)=12.09	ns
Ucp1	ns	ns	ns
Cox4i1	ns	F(1,23)=18.83	F(1,23)=19.29

[†]Two-way ANOVA

Table S4. Interaction and effects of prenatal diet and sex on the gene expression of *Pparα* and *Pparγ* (see Figure 6)[†]

**Liver**	**Interaction**	**Prenatal diet**	**Sex**
Pparα	F(1,22)=7.92	ns	F(1,22)=5.69
Pparγ	ns	F(1,22)=5.92	F(1,22)=19.88
**PAT**	**Interaction**	**Prenatal diet**	**Sex**
Pparα	ns	ns	ns
Pparγ	F(1,22)=9.42	F(1,22)=27.48	F(1,22)=17.89

[†]Two-way ANOVA
